# Unexpected collapse of healthy newborn infants: risk factors, supervision and hypothermia treatment

**DOI:** 10.1111/apa.12244

**Published:** 2013-04-30

**Authors:** Nicolas J Pejovic, Eric Herlenius

**Affiliations:** 1 Department of Neonatology, Sachs' Children and Youth HospitalStockholm, Sweden; 2 Neonatal Unit Q2:07, Department of Women's and Children's Health, Astrid Lindgren Children's Hospital, Karolinska InstitutetStockholm, Sweden

**Keywords:** Cellular phone, Hypothermia, Severe ALTE, Skin-to-skin care, Sudden unexpected postnatal collapse

## Abstract

**Aim:**

To determine the occurrence and risk factors of sudden unexpected postnatal collapse (SUPC) in presumably healthy newborn infants.

**Methods:**

All live-born infants during a 30-month period, in five major delivery wards in Stockholm, were screened, and possible cases of SUPC thoroughly investigated. Infants were ≥35 weeks of gestation, had an Apgar score >8 at 10 min and collapsed within 24 h after birth. Maternal, infant, event characteristics and outcome data were collected.

**Results:**

Twenty-six cases of SUPC were found among 68 364 live-born infants, an incidence of 38/100 000 live births. Sixteen of these cases of SUPC required resuscitation with ventilation >1 min, and 14 of these remained unexplained (21/100 000). Fifteen of the 26 children were found in a prone position, during skin-to-skin contact, 18 were primipara, and 13 occurred during unsupervised breastfeeding at <2 h of age. Three cases occurred during smart cellular phone use by the mother. Five developed hypoxic–ischaemic encephalopathy (HIE) grade 2, and 4 underwent hypothermia treatment. Twenty-five infants had a favourable neurological outcome.

**Conclusion:**

SUPC in apparent healthy babies is associated with initial, unsupervised breastfeeding, prone position, primiparity and distractions. Guidelines outlining the appropriate monitoring of newborns and safe early skin-to-skin contact should be implemented.

## Introduction

Sudden unexpected postnatal collapse (SUPC) of apparently healthy infants in the maternity ward within the first day of life has received increased attention. SUPC includes both severe apparent life-threatening event (ALTE) and sudden infant death (SID) occurring <24 h, in infants where the postnatal adaptation appears normal (10-min Apgar score >8). As SUPC by definition is unexpected, infants with well-known risk, for example prematurity (<35 weeks of gestation), perinatal asphyxia or congenital malformations, are not included in most reports. A majority of reported events occur within 2 h of birth, often at the time of the first breastfeeding attempt 1,2. Even if considered rare, the consequences are grave, with death reported in half of the cases and permanent disability in a majority of the surviving infants 3–5. Initial reports concerning the incidence of early neonatal sudden death (12/100 000 births) and severe early ALTE (12/100 000 births) investigated the occurrence of sudden cardiovascular collapse between 6 and 100 h after birth in infants considered healthy at birth 6. Subsequent reports have used varying inclusion and exclusion criteria for SUPC making estimations of incidence somewhat difficult to compare. Single centres report an incidence of 27–40 SUPC cases per 100 000 births, but the inclusion criteria have been <12 and <72 h postnatal age, respectively 5,7, while recent guidelines for investigations of SUPC include infants with sudden unexpected collapse during the first week of life 8. Several case reports from French maternity wards are available; two of these reports describe their estimation of the incidence of SUPC to be 3.2–3.6/100 000. However, these studies have only included cases occurring within 2 h after birth 3,9–11. Recent national surveys from the UK and Germany have used different criteria for inclusion; SUPC is defined as occurring within the first 24 postnatal hours and excluding (Germany) respectively including (UK), identified and potentially preventable causes 2,4. Both these national surveys report an incidence of about 3 cases per 100 000 infants (2.6 and 3.5/100 000, respectively).

**Key notes**

- Incidence of sudden unexpected postnatal collapse in three Stockholm University Hospitals was found to be 38/100 000, ten times higher than expected from UK and German national surveys.
- Prone, asphyxiating position, unsupervised breastfeeding during first 2 h, primiparity but also distractions – such as smart cellular telephone use emerge as risk factors.
- Improved information, active surveillance as well as rapid hypothermia treatment in severe cases are suggested.

A cluster of sudden neonatal collapses was identified during 2010 in one of Stockholm's delivery and neonatal centres and prompted an investigation of the incidence and underlying risk factors of SUPC. The aim of this study is to report the occurrence and risk factors of SUPC in presumably healthy newborn infants in Sweden and more specifically the Stockholm region.

## Method

SUPC in apparently healthy newborns born >35 weeks of gestation was investigated during a 2.5-year period in five major delivery wards and neonatal centres in Stockholm, Sweden (Sachs' Children Hospital, Karolinska University Hospital at Solna, Huddinge & Danderyd and BB Stockholm at Danderyd's Hospital). Around a quarter of all births in Sweden occur in this group of five delivery wards. All children born in this region have clinical data and an ICD-10 diagnosis recorded in a common perinatal chart (Obstetrix®, Siemens). Automated surveys using specific diagnostic criteria are generated once a year to ensure the quality of medical services. A cluster of sudden unexpected neonatal collapses was identified during 2010 in one centre. Subsequently, the perinatal charts of all children born during the period January 1, 2010, to June 6, 2012, with diagnosis suggesting collapse were examined in this and four additional centres.

International Classification of Diseases – Tenth Revision (ICD-10) codes reviewed were as follows: P28.2 cyanotic attack of newborn, P91.61 mild hypoxic–ischaemic encephalopathy (HIE), P91.92 moderate HIE, P91.63 severe HIE, P28.4 other apnoea of newborn and P21.9 birth asphyxia, unspecified. Infants >35 weeks of gestation, with good postnatal adaption (10 min Apgar score >8), and aged 0–24 h, with sudden unexpected collapse, who underwent resuscitation and neonatal unit admission were eligible. Potential cases were identified, and all medical journal data including examinations, diagnostic and therapeutic data were further investigated to determine inclusion or exclusion. The charts of patients admitted to the NICU (neonatal intensive care unit) were also thoroughly reviewed, including their follow-up notes and interviews with staff involved during the initial event. A comparison with the recent UK and German national surveys was made feasible by dividing the SUPC cases into two categories: (i) severe SUPC where no aetiology was found, in accordance with the two recent German surveys 1,4, and (ii) collapses where either a plausible aetiology was found, such as congenital abnormality, infection or congenital neurological/metabolic disease, in accordance with the UK survey 2, or where vigorous stimulation, Continuous positive airway pressure (CPAP) or ventilation less than a minute enabled the infant to resuscitate and recover. These SUPC cases have been included in some reports 3 but only mentioned in the national surveys and not included in their estimations of SUPC incidence 1,4. However, they are included in the recent UK consensus guidelines for investigation of SUPC 8 and require extra investigations as well as surveillance and are thus included in the present investigation.

## Results

We identified 26 cases 12 of SUPC in apparently healthy newborn infants >35 weeks of GA among the 68 364 infants born, an incidence of 38/100 000 live births. Fourteen cases fulfilled all Poets et al. criteria for inclusion in their national surveys of deliveries in Germany: normal postnatal adaption with sudden unexpected collapse occurring within 24 h after birth with subsequent need for intensive resuscitation, neonatal intensive care or surveillance where no aetiology was found 1. Tables 1–3 summarize the perinatal data of the SUPC cases fulfilling all the above SUPC criteria. The SUPC incidence, if including only these 14 cases, calculated on 68 364 live-born infants is 20.5/100 000. All mothers were healthy and had normal deliveries. One mother had a pacemaker but no cardiac symptoms, and one mother had a BMI over 30 (BMI 31). The SUPC events and outcomes are summarized in Tables 2 and 3. Three of the patients were found hypoxic on the mother's breast, while she was using her ‘smart’ cellular phone. Six patients developed HIE (hypoxic–ischaemic encephalopathy) and four underwent hypothermia treatment. These four patients met our neurological criteria for hypothermia treatment: HIE grade 2 or seizures within 6 h after asphyxia. In two of the cases, the cerebral depression was so severe that mechanical ventilation was needed after resuscitation. This off-protocol treatment was initiated after parental consent. The SUPC cases that underwent hypothermia treatment as well as the cases that were associated with distraction by smart phone are briefly described in supplementary material online. See tables for a summary of the events.

**Table 1:** Perinatal characteristics and outcome of 14 cases of SUPC <24 h: Pregnancy and birth

Case	1	2	3	4	5	6	7	8	9	10	11	12	13	14
APGAR	9/10/10	6/9/10	10/10/10	8/10/10	9/10/10	9/10/10	9/10/10	10/10/10	9/10/10	9/10/10	9/10/10	9/10/10	9/10/10	7/8/10
Gravidity	1/1	4/4	1/1	1/1	1/1	1/1	2/2	1/1	1/1	1/1	1/1	3/3	2/2	1/1
Smoking	No	No	No	No	No	Yes	No	No	No	No	N/A	N/A	N/A	N/A
Maternal drugs	Epi Oxy	Oxy	Epi Oxy	NO_2_ Oxy	Epi	NO_2_ Oxy	Oxy	Oxy	Oxy		NO_2_	NO_2_	NO_2_	NO_2_
Type of delivery	VD	VD Ind	VD	VD	VD	VD	VD Ind	VD	VD	ES	VD	VD	VD	VD
Gender	Male	Male	Male	Male	Female	Female	Male	Male	Female	Female	Female	Female	Male	Female
Umbilical artery pH at birth	7.28	N/A	7.23	7.22	7.26	N/D	7.43	7.42	7.21	N/D	7.24	N/D	N/D	7.05
Birth weight	3735	3960	4220	4580	3915	2575	2855	3300	3180	3190	2525	4514	2812	3490
Gestational age	41 + 0	39 + 4	39 + 4	40 + 1	41 + 1	38 + 2	36 + 0	39 + 3	40 + 1	38 + 2	35 + 6	41 + 3	42 + 1	38 + 2

SUPC, Sudden unexpected postnatal collapse; ES, elective Caesarean section; N/D, not done; VD, Vaginal delivery.

**Table 2:** Perinatal characteristics and outcome of 14 cases of SUPC <24 h: Neonatal event

Case	1	2	3	4	5	6	7	8	9	10	11	12	13	14
Age at event	90 min	120 min	120 min	60 min	20 h	90 min	60 min	60 min	15 h	13 h	3 h	15 min	30 min	120 min
Last caretaker	Mother	Mother	Mother	Mother	Mother	Mother	Mother	Mother	Parents	Parents	Mother	Mother	Mother	Mother
Age when last put to breastfeed	At event	At event	At event	At event	Before event	At event	At event	At event	N/A	N/A	At event	N/D	N/D	At event
Mother asleep	No	No	No	No	Yes	No	No	No	No	N/A	N/A	No	No	No
Found by	Father	Midwife	Midwife	Father	Parents	Midwife	Father	Midwife	Parents	Parents	Midwife	Midwife	Midwife	Midwife
Location	DS	DS	DS	DS	Maternity	DS	DS	Maternity	Maternity	Maternity	Maternity	DS	DS	DS
Time at event	21:30	22:30	13:00	17:00	22:00	22:20	19:50	14:20	11:00	01:00	18:30	05:15	03:00	19:00
Place found	Mother	Bed	Mother	Mother	Bed	Mother	Mother	Mother	N/A	N/A	Mother	Mother	Mother	Mother
Face covered by mothers breast	Yes	Yes	No	No	Yes	Yes	Yes	Yes	N/A	N/A	Yes	Yes	Yes	Yes
pH min after event	7.1	6.76	7.26	6.88	N/D	7.06	N/D	7.1	N/D	N/D	6.83	6.8	N/D	7.17
Mobil phone distraction	Yes	Yes	No	No	No	No	No	No	No	N/A	Yes	N/A	No	N/A
Cobedding	No	No	Yes	No	Yes	No	No	No	N/A	N/A	N/A	No	No	No
Neurological symptoms	None	HIE 2	HIE 2	HIE 2	HIE 2	None	None	None	No	No	HIE 1	No	HIE 2	No
Hypothermia treatment	No	Yes	Yes	Yes	Yes	No	No	No	No	No	No	No	No	No
Hypoglycaemia	No	No	No	No	No	No	N/D	2.4 gr/L	No	No	No	No	No	No
Bag and mask ventilation	Yes	Yes	Yes	Yes	Yes	Yes	Yes	Yes	Yes	Yes	Yes	Yes	Yes	Yes
CPAP	No	Yes	Yes	Yes	Yes	Yes	No	Yes	No	No	Yes	Yes	Yes	Yes
Mechanical ventilation	No	Yes	No	No	Yes	No	No	No	No	No	No	No	Yes	No
Brain ultrasound	N/D	Normal	Normal	Normal	Normal	N/D	N/D	N/D	No	N/D	Normal	No	Oedema	N/D
EEG on admission	N/D	Path	Path	Path	Path	N/D	N/D	N/D	N/D	N/D	Normal	N/D	Path	N/D
Echocardiography	N/D	Normal	Normal	Normal	Normal	N/D	Normal	N/D	N/D	N/D	N/D	N/D	PPHN	N/D
Multichannel cardiorespiratory recording	N/D	N/D	N/D	N/D	N/D	N/D	N/D	Normal	Immature	N/D	N/D	N/D	N/D	N/D
Infectious and metabolic investigation	Normal	Normal	Normal	Normal	Normal	N/D	N/D	N/D	Normal	N/D	Normal	Normal	Sepsis	Normal

DS, delivery suite; N/D, not done; SUPC, Sudden unexpected postnatal collapse; CPAP, continuous positive airway pressure; Ind, induction; VD, Vaginal Delivery, PPHN, persistent pulmonary hypertension; N/A, not available.

**Table 3:** Perinatal characteristics and outcome of 14 cases of SUPC <24 h: Discharge/follow-up

Case	1	2	3	4	5	6	7	8	9	10	11	12	13	14
Neurological status upon discharge	Normal	Hypotonia	Hypotonia	Normal	Normal	Normal	Normal	Normal	Normal	Normal	Normal	Normal	Hypertonia	Normal
EEG on Discharge	N/D	Path	Path	Normal	N/A	N/D	N/D	N/D	Normal	N/D	N/D	N/D	N/D	N/D
MRI	N/D	Normal	Normal	Normal	Normal	N/D	N/D	N/D	N/D	N/D	N/D	N/D	Normal	N/D
Neurological status on follow-up	N/D	Normal EEG ok	Normal EEG ok	CP	Normal	N/D	N/D	N/D	Normal	N/D	N/D	Normal	Normal	N/D

CP, cerebral palsy.

Twelve additional potential SUPC cases were identified in presumably healthy infants; these are summarized in Tables 4–6. All of these twelve infants fulfil the inclusion criteria established in ‘The guidelines for the investigation of newborn infants who suffer a sudden and unexpected postnatal collapse’^8^. In these 12 SUPC cases, vigorous stimulation or short ventilation (<1 min) enabled infants to recover (n = 9), or a plausible aetiology was found (n = 3, Tables 4–6).

**Table 4:** Perinatal characteristics and outcome of SUPC <24 h stimulation or ventilation <1 min or potential aetiology discovered: Pregnancy and birth

Case	15	16	17	18	19	20	21	22	23	24	25	26
APGAR	9/10/10	9/9/9	9/10/10	9/10/10	9/10/10	9/10/10	9/10/10	9/10/10	7/10/10	6/9/10	9/9/10	7/8/9
Gravidity	1/1	3/3	2/2	1/1	1/1	1/1	1/1	2/2	1/1	2/2	1/1	1/1
Smoking	No	No	No	No	Yes	No	No	No	N/A	No	No	No
Maternal drugs	NO_2_	NO_2_	Epi NO_2_	Epi NO_2_	Epi NO_2_ SSRI	NO_2_	Epi NO_2_	NO_2_ SSRI	Insulin	Epi Oxy	NO_2_	Oxy DS
Type of delivery	VD	VD	VD Ind	VD	VD	VD	VD	VD	AS	AS	VD	AS
Gender	Female	Male	Male	Male	Female	Male	Female	Female	Male	Female	Female	Male
Umbilical artery pH at birth	7.28	7.26	7.06	7.31	7.26	7.22	7.24	7.25	7.13	7.15	7.37	7.17
Birth weight g	3106	4150	4095	3210	2925	2855	2930	3795	3470	3315	3240	3110
Gestational Age	39 + 4	41 + 0	40 + 3	38 + 5	37 + 1	37 + 0	37 + 6	38 + 0	36 + 6	40 + 0	38 + 6	41 + 4

AS, acute section; Epi, Epidural; Ind, induction; VD, Vaginal delivery.

**Table 5:** Perinatal characteristics and outcome of SUPC <24 h stimulation or ventilation <1 min or potential aetiology discovered: Neonatal event

Case	15	16	17	18	19	20	21	22	23	24	25	26
Age at event	45 min	8 h	12 min	120 min	16 h	1 h + 24 h	60 min	3 h	30 min	15 h	3 h	60 min
Last caretaker	Mother	Mother	Midwife	Mother	Mother	Mother	Mother	Mother	Grandfather	Mother	Mother	Mother
Age when last put to breastfeed	At event	N/A	N/D	At event	At event	At event	At event	At event	N/D	At event	At event	At event
Mother asleep	No	No	No	No	No	No	No	No	No	No	No	No
Found by	Midwife	Mother	Midwife	Mother	Parents	Mother	Mother	Midwife	Midwife	Midwife	Mother	Midwife
Location	DS	DS	DS	DS	Maternity	DS	DS	Maternity	DS	Maternity	DS	DS
Time at event	18:00	04:05	03:40	21:00	21:00	13:00	05:00	23:00	13:00	03:00	12:00	13:00
Place found	Mother	Mother	Mother	Mother	Mother	Mother	Mother	Mother	Grandfather	Mother	Mother	Mother
Face covered by mothers breast	N/A	No	No	Yes	Yes	Yes	Yes	Yes	No	No–Yes–No	Yes	No
pH min after event	N/D	N/D	N/D	N/D	N/D	N/D	N/D	N/D	N/D	N/D	7.2	7.3
Mobil phone distraction	No	No	No	No	No	No	No	No	No	No	No	No
Cobedding	No	No	No	No	No	No	No	No	No	No	No	No
Neurological symptoms	No	No	No	No	No	No	No	No	HIE 1	No	No	No
Hypothermia treatment	No	No	No	No	No	No	No	No	No	No	No	No
Hypoglycaemia	No	No	No	No	No	No	No	No	No	No	No	No
Bag and mask ventilation	No	Yes	Yes	No	No	No	No	No	No	No	Yes	Yes
CPAP	Yes	Yes	Yes	Yes	No	No	No	No	Yes	No	Yes	Yes
Mechanical ventilation	No	No	No	No	No	No	No	No	No	No	No	No
Brain ultrasound	N/D	N/D	N/D	N/D	N/D	N/D	N/D	No	Normal	Normal	N/D	Normal
EEG on admission	N/D	N/D	N/D	N/D	N/D	Normal	N/D	N/D	N/D	Path	N/D	No
Echocardiography	N/D	N/D	N/D	N/D	N/D	N/D	N/D	PPHN	Normal	Normal	Normal	Normal
Infectious and metabolic investigation	Normal	N/D	CRP 93	N/D	N/D	P-glu 2,4	N/D	Normal	Thrombocytopenia Hypoglycaemia	Normal	Normal WLD	Tracheoesophageal fistula and E. atresia

N/D, not done; N/A, not available; DS, delivery suite; CPAP, continuous positive airway pressure; WLD, Wet Lung Disease.

**Table 6:** Perinatal characteristics and outcome of SUPC <24 h stimulation or ventilation <1 min or potential aetiology discovered: Discharge/follow-up

Case	15	16	17	18	19	20	21	22	23	24	25	26
Neurological status upon discharge	N/D	N/D	N/D	N/D	N/D	N/D	N/D	Normal	Normal	Normal	Normal	Normal
EEG on discharge	N/D	N/D	N/D	N/D	N/D	N/D	N/D	N/D	N/D	Normal	N/D	N/D
MRI	N/D	N/D	N/D	N/D	N/D	N/D	N/D	N/D	N/D	N/D	N/D	N/D
Neuropsychomotor develop follow-up	N/D	N/D	N/D	N/D	N/D	N/D	N/D	N/D	N/D	N/D	N/D	Normal
Neurological status on follow-up	N/D	N/D	N/D	N/D	N/D	N/D	N/D	N/A	N/A	Normal	Normal	Normal
Other findings on follow-up	N/A	N/A	N/A	N/A	N/A	N/A	N/A	N/A	N/A	N/A	Normal	Normal

Case 17: Suspected infection but no bacteria or virus found in repeated blood and CSF samples. Case 24: EmCD due to MSAF and scalp lactate 4,8 Two unexpected collapses and a third one requiring admission Slight transient pathology on EEG but brain ultrasound was normal. Case 25: A collapse requiring >3 min of minute ventilation, but X-ray revealed wet lung disease (WLD). Case 26: 4-min ventilation required, but a tracheoesophageal fistula explained the sudden and unexpected collapse of this infant.

N/D, not done; N/A, not available.

In nine of these infants, vigorous stimulation, CPAP or ventilation <1 min with bag and mask led to rapid recovery and healthy infants. One developed cyanosis and was found in poor condition but not lifeless. Sepsis (CRP 93) was identified as a plausible causative factor behind the sudden deterioration, even if no agent was identified with virological as well as bacteriological blood culturing (case 17). He was treated accordingly including antibiotics and did not suffer neurological distress. One child required >3-min ventilation and developed wet lung disease and required two weeks with CPAP support at the NICU (case 25). Finally, an infant considered healthy at birth collapsed after 1 h during feeding and required 4 min of ventilation before temporarily recovering, and a tracheoesophageal fistula and oesophagus atresia were discovered, likely causing the sudden collapse (case 26).

Of the 26 mothers, one smoked and three had fluoxetine prescribed during pregnancy. Twenty-two SUPC infants had normal vaginal deliveries (PN), and one was delivered by elective Caesarean section (ES) and three by acute Caesarean section (AS). In 8 of the 26 cases, the mother had epidural anaesthesia. Only one event occurred while the parents were asleep. Of the 26 SUPC cases, 18 were born to primiparous mothers, 13 were male, and 17 collapses occurred during the first 2 h. In 15 of the 23 SUPC cases, where information was available, the infant's face was covered by their mother's breast. Five more SUPCs requiring extensive NICU care were discovered in the present investigation, but all events had occurred after 24 h (36–116 h postnatal age) and were therefore excluded from the present summary and Tables.

## Discussion

We report 26 cases of early neonatal collapse, an incidence of 38/100 000 live births, and 4 underwent hypothermia treatment with a favourable outcome.

### SUPC incidence and definitions

The incidence of SUPC has been estimated to range between 2.6–5/100 000 live births 2,13. However, clusters of cases have previously been described, with a similar incidence as the present report 5,7. Most publications indicate that the published estimations of SUPC are lower than what occur in the wards and only reflect the most critical events 4. Notably, no consensus exists for coding unexpected postnatal collapse in ICD 10, and this very possibly contributes to it being under reported. Events with rapid and favourable outcome could therefore easily be missed in large surveys 3. SUPCs where vigorous stimulation enabled recovery are included in reports by Andres et al. (2 of 6 infants) 3 and Grylack et al. (20 infants) 14 but only mentioned in other studies*,* for example Poets et al. 1. Notably, early ALTE cases that recover after vigorous stimulation are also included in the recently published guidelines for investigation of SUPC 8. We think that including the sudden collapse in a newborn where vigorous stimulation, CPAP or less than 1-min ventilation was successful in the SUPC definition is valid for several reasons. Potentially, these cases could have devastating consequences if discovered later. They also should be investigated for underlying causes of the collapse. Including these cases increases the incidence of unexpected collapse in apparently healthy newborn in the present population to 38/100 000 infants. Thus, the current report indicates that, even if still rare, SUPC might occur more frequently than that indicated in recent surveys and that the risks and factors contributing to it need to be recognized in the delivery wards.

The definitions of when (<2–<72 h or 7 days) and at what gestational age (>week 35 or >week 38) unexpected collapses of apparent healthy infants occur differ between published reports 1–4,7,15,16. A consensus regarding the definition of SUPC would facilitate the comparison between studies and enable the paediatric and obstetric community to write evidence-based guidelines on risk factors and postnatal surveillance even in infants considered healthy at birth. The initial German national survey included infants born >37 gestational weeks 4 but altered inclusion criteria the following year to also include infants born >35 gestational weeks 1. This is in accordance with the age definition that was established in the UK consensus guidelines concerning investigations of SUPC 8. Also, apparently healthy infants born after gestational week 35 + 0 in maternity wards are under parental supervision only, in a number of centres. The risk of an unexpected cardio-respiratory collapse is small, but the outcome, potentially catastrophic. An awareness of and guidelines regarding adequate supervision of newborns especially during the first hours of postnatal life should be implemented.

A weakness of the present study is that it is a retrospective review of patient journals, using primarily charts and investigations. Strengths of the current study are that we have had access to all patient charts in a common digitalized database. The authors were directly involved in some of the cases (8) and were able to personally interview involved personnel if clarifications were needed in single cases.

### Hypothermia treatment

This is also the first report suggesting that hypothermia treatment could improve outcome after SUPC. A review of 17 and 45 cases in Germany and United Kingdom national surveys showed a mortality rate of 42% and 27%, respectively 2,4. 60% of the cases were neurologically abnormal at discharge in the German study 4, while a third died or had abnormal neurological outcome in the UK study 2. Higher mortality is reported on a small number of patients in France 3,10. No infants died in the present cohort. This could be due to good monitoring practices and cases found at an early stage. The hypothermia treatment of the 4 patients was successful, and follow-up has been normal in 3 of 4 cases. One case was considered to have a normal outcome until a mild cerebral palsy was detected at follow-up at 24 months (Table 3). Despite that these were severe SUPC cases, outcome so far (up to 2 years postevent) is excellent. This should encourage further studies of the effect of hypothermia on postnatal ischaemic events.

### Risk factors: position/distractions

The transition from foetal to extrauterine life could make the newborn more vulnerable during the first hours of life. The initial surge of adenosine and prostaglandins during 17 and catecholamines after birth 18 is followed by a period of diminished responsiveness to external stimuli 4 and increased vagal tone 19. Prone position, first breastfeeding attempt, cobedding, mother in episiotomy position, primiparous mother and parents left alone with baby during first hours after birth have been identified as risk factors for SUPC or SID in several recent publications 1–3,5,10,11,13,16. Our data are in accordance with this (see Tables 1–6).

Recommendations that all newborn infants should be placed in supine position within the first few hours after birth are emphasized in these reports as well as in the recently updated guidelines from the American Academy of Pediatric (AAP) regarding a safe infant sleeping environment to reduce the risk of SID 20.

Three cases involved breastfeeding mothers who were using their mobile phones in the absence of other caregivers and the staff subsequently discovered collapse of child. Informal reports from midwives suggest that the extensive use of smart/mobile phones, messaging and social networking after delivery is an emerging trend, with some mothers writing up to 30 messages during the first 2 h after delivery.

Smart phones, especially when used for text messaging, have already been linked to an increasing amount of traffic accidents 21,22. The visual–manual interaction during social networking and text messaging is a powerful distraction during driving and has been linked to a 23-fold increase in the risk of traffic accident, comparable to having 1.5 mg/mL alcohol in the blood 22. If smart phones distract and increase accidents while driving a car, it is plausible that they could be a risk factor for SUPC. Thus, at least one caregiver should be focused on the baby during the first postnatal hours especially during prone skin-to-skin contact.

Postnatal bonding strengthens mother and child relationship. However, initiating breastfeeding is demanding for both the newborn and the inexperienced mother. Skin-to-skin care is encouraged in most modern delivery units, but midwives should closely assist the mother and monitor the position of the newborns as well as their airways 13. Cognitive and emotional distraction combined with parasympathetic mediated allostasis and increased vulnerability for airway obstruction during the first breastfeeding attempts could be a new risk factor for SID and S-ALTE and should be further investigated. Early skin-to-skin care exhibits higher rates of breastfeeding and possible emotional benefits. It is encouraged in most OECD countries (Organization for Economic Co-operation and Development) and appears to be safe, with ‘no apparent short- or long-term negative effects' 23. However, in Spain, SUPC increased from 0.06/1000 to 0.74/1000 live births after introduction of early skin-to-skin care (SSC) 24,25. Introducing parental information and supervision routines did not reduce the number of SUPC, but subsequent cases were less severe. We are not aware of any studies comparing the practice of SSC between different delivery units. However, the rate of early SSC in the NICU is higher in Sweden than in other Nordic countries 26. We should be careful comparing data from previous studies, but skin-to-skin contact, prone position and primiparity are associated with the majority of SUPC cases reported 1,4,16,24,25. The high rate of SUPC in this report could possibly be linked to widespread introduction of early SSC in our units. Alleged risk factors such as using smart/cellular phone during breastfeeding were identified in three cases. Male gender is a risk factor for SUDI (occurring between 1 week and 1 year) but has not emerged as a risk factor for SUPC (occurring at 0–24 h) in national surveys 1. That 13 of 26 infants were male in the present report suggests that gender is not a risk factor in this population study. A cluster of events, early skin-to-skin care, new risk factors inclusion of less critical events, where infants recovered, as well as direct access to all patient charts and caregivers, could explain the present tenfold increase in the incidence of SUPC compared with the UK and German national surveys.

### Investigation of SUPC cases

The occurrence of SUPC events (S-ALTE and SID) should be investigated according to newly published guidelines 8. These guidelines cover sudden unexpected postnatal collapse in the first week of life in term and near-term babies (>35 weeks of gestation), complementing the guidelines for investigation of sudden unexpected death in infancy (SUDI) that emphasize deaths outside the hospital 27. Structured investigations, to determine the underlying cause as congenital anomalies, metabolic and infectious disease, are a requirement for treatment. Moreover, in cases where no aetiology is found, a structured investigation can help in the counselling and to determine the appropriate level of surveillance. However, only 5 of the 12 cases presented in Tables 4–6 underwent the recommended investigations, and the structure of follow-up differed between the five investigated wards. National guidelines for follow-up of premature infants exist, and we will try to imply guidelines for prevention as well as follow-up also for SUPC.

In 20 SUPC or ALTE events, where multichannel recordings of cardiorespiratory function were performed, 12 had apnoeas greater than 15 sec and four exhibited bradycardia 14. This indicates that an increased presence of autonomic instability compared with control term infants might be present in the infants with SUPC and make them more vulnerable. In the present study, only two infants underwent multichannel cardiorespiratory recordings and one exhibited, for the age, an immature cardiorespiratory control (case 9). Possibly including plethysmograph cardiorespiratory recording could be considered in the investigation of SUPC in surviving infants. In this way, potentially treatable causes of collapse could be found at early stage and the need of monitoring assessed. The counselling of parents and planning of follow-up may also be improved.

### Preventive measures

The emergent pattern of risk factors for SUPC differs partly from SIDS and should help us to formulate guidelines for parental information and postnatal surveillance that are compatible with safe newborn care. The concept of safe early skin-to-skin contact (S-SSC) could be a step to prevent the S-ALTE and possible SID events during the first 24 h of life. Even if cases are rare in large populations, the benefits of such practice should be evaluated. Introducing S-SSC in delivery rooms could include the following: checklists for midwives, delivery of oral and written information to parents, schedule for monitoring of infants' condition in particular 0–3 h postpartum 4,13, observation of first breastfeeding attempts 1 and reducing risk of distractions. Regular information campaigns and audits should keep staff updated. Unobtrusive wireless heart rate monitors are a technology that has interesting potentials, but an evaluation of the potential benefits of electronic monitoring to help clinical staff during the first neonatal hours in thousands of healthy newborns is needed 28. S-SSC integrates the benefits of early parental bonding with safe breastfeeding practice and gentle monitoring of the potentially vulnerable newborn.

## Abbreviations

SUPC: Sudden unexpected postnatal collapse
S-ALTE: Severe apparent life-threatening event
SID: Sudden infant death

## SUPPORTING INFORMATION

**Data S1.** Unexpected collapse of healthy newborn infants: risk factors, supervision and hypothermia treatment.
