# Supplementary material for: Unexpected collapse of healthy newborn infants: risk factors, supervision and hypothermia treatment
Source: Acta Paediatr. 2013 Apr 30;102(7):680–8. doi: 10.1111/apa.12244 (PMC3709122; doi:10.1111/apa.12244)
Supplement: Supplementary file 1 [file apa0102-0680-SD1.docx]

**SUPPORTING INFORMATION ONLINE;**

**Unexpected collapse of healthy newborn infants: risk factors, supervision and hypothermia treatment**

**Case 1**

A boy was found 90 min after birth by father at mother’s breast while she was using her smart/cellular phone. The baby was cyanotic, hypotonic, did not breathe and had bradycardia 70-80/min. He was resuscitated with bag and mask. He was monitored for signs of HIE but the neurological examinations as well as laboratory, infectious investigations remained normal and he was dismissed from the neonatal ward and surveillance after 5 days. Extended neurodevelopmental follow up was not deemed necessary but routine follow up controls have all been normal.

**Case 2**

A boy was found lifeless by the midwife at mother’s breast while she was using her smart/cellular phone. He did not recover spontaneous breathing after extensive resuscitation. He developed HIE grade 2 with seizures. Off-protocol hypothermia treatment was given for 72h. He remained on mechanical ventilation for 36 h. Extensive investigation identified no underlying condition. On follow up at 1 year he has a normal neuropsychomotor development.

**Case 3**

A boy was lying supine between resting parents when found cyanotic by midwife during routine control. He was resuscitated and developed HIE 2. Off-protocol hypothermia treatment was given for 72h. Extensive investigation identified no underlying condition. At follow up at 1 year he has a normal neuropsychomotor development.

**Case 4**

Father found the boy lifeless at mother’s breast 60 min after birth. He was put on mechanical ventilation after extensive resuscitation. He developed HIE 2 with seizures and underwent off-protocol hypothermia treatment during 72 h. Extensive investigation identified no underlying condition. At 12-month he had a normal neuropsychomotor development. A mild spastic cerebral palsy was recognized at 24-month follow up.

**Case 5**

A girl was lying supine between both sleeping parents and found cyanotic when they woke up. She was resuscitated and subsequently put on mechanical ventilation because of repeated apnea. She developed HIE 2 with seizures and underwent successful off-protocol hypothermia treatment during 72h. Plethysmographic cardiorespiratory recording was normal, extensive investigation identified no underlying condition. She was slightly hypotonic at discharge but able to breast feed. Examination at 10 month revealed no abnormality in neuropsychomotor development.

**Case 6**

A girl was laying prone breastfeeding while her mother was in the lithotomy position. Mother had BMI 30. The baby was found cyanotic, hypotonic without breathing movements and had bradycardia <100. She was resuscitated with bag and mask. She recovered promptly. She was admitted to the neonatal intensive care unit, but surveillance was performed in the neo-maternity ward next door to the NICU. Cardiorespiratory, infectious and metabolic investigations did not find any underlying condition. Extensive follow up was not deemed necessary but routine controls have all been normal.

**Case 7**

A boy was found 1 hour after birth lying prone at mothers breast hypotonic and cyanotic. Immediate resuscitation with bag and mask commenced with rapid recovery. Initial 30-minute CPAP treatment and subsequent 24 hours surveillance at the neonatal ward with normal outcome. ECG and cardiac echocardiography, laboratory investigations as well as neurological status were all normal. Benign hyperbilirubinemia after 48 hours, normal outcome with no apparent neurodevelopmental sequela at follow up.

**Case 8**

A boy was lying prone on the mother’s breast 1 hour after birth while she was in the lithotomy position. The baby was silent for approximately 3 minutes and found hypotonic and cyanotic without breathing movements. He was resuscitated with bag and mask and recovered breathing after 5 minutes. His neurological as well as general physical and laboratory examination was normal but he required CPAP and surveillance at the neonatal ward during the following 24 hours. Plethysmographic cardiorespiratory recordings during first day as well as 4 months of age were both adequate for his age. Extended neurological follow-up was not deemed necessary.

**Case 9**

A girl was found cyanotic by her parents 15 hours after a normal delivery. They saw no chest movements and rushed her to the Pediatrician who was doing rounds next door. She had hypotonia, apnea, bradycardia (<60/min) girl and was resuscitated using supplementary oxygen, ventilation and heart compressions. All investigations were negative during 2 days of surveillance at the NICU. Due to repeated episodes of apnea and cyanosis during first week she was admitted to the pediatric unit for surveillance and extended investigations. Multichannel cardiorespiratory monitoring showed several episodes of irregular breathing and short (max 11 s) apneas with concomitant desaturation and short bradycardia that was interpreted as immature cardiorespiratory control.

**Case 10**

A Girl was found by parents cyanotic and apneic 13h after delivery. She was ventilated >1 min, then vomited clear mucous secretions before spontaneous breathing resumed. She was suctioned and put on oxygen because of inadequate saturation, but continued to have apneoic episodes and minor vomiting. She was brought to NICU for surveillance. Routine investigations were normal, but cardiac echography and EEG were not performed. Extensive follow up was not deemed necessary but routine controls have all been normal.

**Case 11**

A girl born after normal delivery at 35+6 weeks was considered healthy by the midwife and had no extra surveillance. The mother was left alone. At 3 hours of age the midwife found the girl lifeless at mother’s breast while she was using her smart/cellular phone. Apgar during resuscitation was 4, 5, 5 and pH 6.83, BE -18 (mmol/L). Five ml Tribonate^®^ was given after 15 min resuscitation. After 20 minutes of ventilation the girl vomited and recovered spontaneous breathing. She was put on CPAP at the NICU was irritable. Cerebral ultrasound showed oedema. The EEG at admission was pathological but had no seizures. She developed HIE 1 and did not fulfill all criteria for hypothermia treatment. Laboratory investigations identified no underlying condition and EEG was normal at discharge.

**Case 12**

A boy was born after a rapid, but normal vaginal delivery and considered healthy by the midwife (Apgar 9, 10, 10). The umbilical blood gas was not taken at birth. At 15 min of age, the mother thought that the girl “fell asleep” but did not see chest movements. The staff found the baby cyanotic, hypotonic and bradycardic (<60/min). Resuscitation was started immediately. The hemoglobin was 201 (g/l) and pH 6,8, pCO_2_13,6, BE-20 (mmol/L) on blood gas. She recovered spontaneous breathing after thirty minutes of ventilation and was transferred to NICU. She had supplemental oxygen and CPAP during first 12 hours for adequate saturation. All investigations were normal except transient tachypnea of newborn during first day. Normal outcome with no apparent neurodevelopmental sequela at follow up.

**Case 13**

A boy was born at 42+1 weeks after normal delivery. He was considered healthy, but at 35 minutes of age suddenly developed progressive cyanosis with faint grunting breathing. He deteriorated rapidly with cardiorespiratory collapse and bradycardia (<70/min) within 2 minutes. Resuscitation lasted one hour, including 8 minutes of heart compressions and 12 ml of Tribonate^®^. He was intubated and put on mechanical ventilation. He was planned for ECMO but improved after NO and inotropic support. A pneumothorax, possibly due to extended resuscitation efforts, was found. Echocardiography exhibited signs of secondary pulmonary hypertension. Brain ultrasound shows no bleeding, but signs of oedema and collapsed lateral ventricles. EEG and aEEG monitoring was pathological. He had no typical seizures but was put on phenobarbital because of twitching and repetitive chewing. He received 10 days of antibiotic treatment due to suspected infection with elevated CRP (max 145 at 48 hour postnatal age) and elevated pro-calcitonin, but all infectious investigations were negative. Extubation to CPAP was possible after 10 days of mechanical ventilation and was continued for more days. He developed renal hypertension due to temporary acute tubular necrosis induced by asphyxia. His renal function normalized after 6 months of antihypertensive treatment. He had hypertonic extremities at one month of age, but his neuropsychomotor development is within normal range at the 2-year follow-up.

**Case 14**

A girl was born after normal delivery. The amniotic fluid was meconium stained and girl was considered healthy by the midwife. At 2 hours of age she was found cyanotic in prone position with the face covered by mothers breast. She was bradycardic and immediately rescuscitated. The girl recovered after 2 minutes ventilation and was transferred to NICU.
